# Flake Graphene as an Innovative Additive to Grease with Improved Tribological Properties

**DOI:** 10.3390/ma15217775

**Published:** 2022-11-04

**Authors:** Małgorzata Djas, Anna Matuszewska, Beata Borowa, Krystian Kowiorski, Piotr Wieczorek, Marcin Małek, Adrian Chlanda

**Affiliations:** 1 Łukasiewicz Research Network—Institute of Microelectronics and Photonics, al. Lotników 32/46, 02-668 Warsaw, Poland; 2 Faculty of Chemical and Process Engineering, Warsaw University of Technology, Waryńskiego 1, 02-645 Warsaw, Poland; 3 Łukasiewicz Research Network—Automotive Industry Institute, Jagiellońska 55, 03-301 Warsaw, Poland; 4 Faculty of Civil Engineering and Geodesy, Military University of Technology, Gen. Sylwestra Kaliskiego 2, 00-908 Warsaw, Poland

**Keywords:** flake graphene, graphene oxide, reduced graphene oxide, lubricants, grease

## Abstract

The paper presents the results of research on the use of flake graphene as an additive to plastic grease in order to improve its tribological properties. The influence of concentration (0.25–5.00 wt.%) and the form of graphene (graphene oxide, reduced graphene oxide) on selected properties of the base grease were investigated. It has been found that the addition of graphene flakes improves the anti-wear properties of the lubricant. The greatest improvement in the properties of the lubricant was achieved by using graphene at a concentration of 4.00 wt.%; the reduction in the average diameter of the wear scar was almost 70% for GO and RGO, compared to the base lubricant without the addition of graphene.

## 1. Introduction

Lubricants, such as oils and greases play a key role in many industries, including the automotive, transportation, chemical and food industries in particular. The factor driving the growth of the market for these products in the world is the dynamic technological development. Lubricant manufacturers have to keep pace with more and more advanced vehicles as well as innovative machines and devices, where precision and smooth operation are crucial for their efficient and profitable operation. During mechanical processes, as a result of strong friction between the surfaces, wear of the mechanical elements of the friction junction occurs and heat is generated, which ultimately reduces the efficiency of work and shortens the life of the device. It is estimated that about 23% (119 EJ) of global energy consumption is due to anti-friction and regeneration of friction elements [1]. The reduction of friction not only contributes to the reduction of energy consumption, but also fuel savings and the related financial and environmental benefits, in particular the reduction of CO_2_ emissions [2]. In addition, conventional additives with anti-wear (AW) and anti-seizure (extreme pressure EP) properties are mainly based on sulfur, chlorine or phosphorus compounds, which are not environmentally friendly [3,4]. Therefore, it is necessary to develop innovative components, modifiers and additives to lubricants with significantly improved tribological properties and reduced negative impact on the natural environment. It has been found that one such additive may be flake graphene, including its derivatives (graphene oxide—GO; reduced graphene oxide—RGO), due to its unique properties, such as thickness of only one or a few carbon layers, layered structure, small flake size. Having in mind flake graphene materials’ thickness, most of them are classified as nanoadditives. Conventional EP and AW additives act by adsorbing active groups to the friction surface or react chemically with the metal surface to form a modified boundary layer. On the other hand, EP and AW nanoadditives can protect frictional mating surfaces mainly through: mending effect, polishing effect, protective film formation mechanism and rolling bearing effect [5,6].

Flake graphene, which is a known 2D carbon nanomaterial, is obtained from graphite. At the same time, it should be emphasized that graphite is characterized with greasing properties and it is widely implemented, i.e., as an anti-wear agent. Graphene is another allotropic type of carbon, next to graphite. GO is the oxidized form of graphene, decorated with oxygen functional groups (e.g., hydroxyl, carboxyl or alkoxy groups). GO particles are highly hydrophilic and form stable dispersions in water and some organic solvents. RGO is obtained by reduction of oxygen functional groups of GO. Contrary to GO, rGO is hydrophobic and show high mechanical strength, large specific surface area and high electric and thermal conductivity [7].

The use of flake graphene is currently the subject of much scientific research in various areas, such as composites [8], functional coatings [9], electronics [10], energy storage [11], sensors [12] and even medicine and tissue engineering [13,14]. One of the intensively developed and promising areas of application of flake graphene is tribology. It was found that the introduction of flake graphene as an additive to the lubricant has a positive effect on its tribological properties, reducing the value of the friction coefficient and the degree of surface wear. Better lubricity is achieved by nanostructured arrangement of graphene flakes and even by graphitization. In addition, the presence of a protective film on the surface of the friction junction elements, arranged parallel to the direction of movement, was found, which indicates that the graphene flakes slide between each other due to friction [15].

As part of the research described in the literature, the tribological properties of various lubricants (lubricating oils, plastic greases) with the addition of various forms of graphene flakes (graphene, graphene oxide, reduced graphene oxide, graphene nanoplatelets) were analyzed. The tribological properties of polyalphaolefin-2 (PAO2) base oil with the addition of multilayer graphene containing 0.05 wt.%, 0.10 wt.% and 0.50 wt.% were investigated using a 4-ball tester. The tests were carried out at loads from 120 N to 400 N and rotational speeds in the range of 100–400 rpm [16]. It was found that, regardless of the tested concentration, the use of graphene in the oil improves the tribological properties (reduction of the friction coefficient) of the oil compared to the oil without the additive, with the best results being obtained for a concentration of 0.05 wt.%. However, in the case of a 10-fold increase in the concentration of graphene in the oil to 0.50 wt.%, the value of the friction coefficient increased, approaching the results obtained for pure oil [16]. The reason for this may be the tendency of the graphene material to agglomerate in the base oils. To prevent this from happening, dispersants can be added to the oil or the graphene can be chemically modified [17,18].

Graphene as an anti-wear additive to plastic greases was also tested. The properties of mineral plastic grease thickened with lithium saponified, enriched with the addition of graphene nanoplatelets with a concentration of 0.50 wt.%, 1.00 wt.%, 5.00 wt.% and 10.00 wt.% were investigated. Various loads were applied: 5 N, 10 N and 15 N [19]. It was found that with an increase in the concentration of graphene in the lubricant, the value of the friction coefficient decreased, in contrast to the results presented earlier, where the best results were obtained for samples containing the lowest concentrations of additives. The value of the friction coefficient decreased by 8.5% for the concentration of 0.50 wt.%, while the increase in the concentration of graphene to 10.00 wt.% resulted in a reduction of the friction coefficient by 16.3% compared to the value of the coefficient obtained for pure lubricant [19]. Fu et al. found that the addition of graphene nanoplatelets to the base grease effectively reduces the value of the friction coefficient and improves the thermal conductivity of the lubricant. The concentration of graphene in the lubricant was 1.00–4.00 wt.%. The best results of the lubricant’s tribological properties were obtained for the graphene concentration in the lubricant of 2.00 wt.% [20], for which the diameter of the friction scar decreased by approx. 14% compared to the base grease.

Flake graphene offers great research opportunities because its surface can be chemically modified and its properties can be controlled in this way. For example, the lubricating properties of monolayer graphene oxide when used as an additive to water-based lubricants were investigated [21]. Tribological tests were performed using a reciprocating sliding configuration at node load 1.88 N. It was found that the addition of GO to the water improved lubrication and provided a very low coefficient of friction of about 0.05 with no apparent wear to the surface after 60,000 cycles of friction tests. It was concluded that the adsorption of graphene oxide on the lubricated surfaces of the friction elements is responsible for the protection of the surface and the material behaves like a protective coating. The change in the composition of the graphene surface, in particular the share of oxygen functional groups, also influences the change in the tribological properties of the graphene material [21]. Wang et al. discovered that in the case of the oxidized form of graphene, the friction between the two surfaces increases with the introduction of epoxy and hydroxyl groups into the molecules. The interaction between the layers is dominated by hydrogen bonds [22]. Liu et al. investigated the effect of two types of graphene, physical graphene produced by liquid-phase exfoliation and graphene produced by chemical oxidation and reduction, on the tribological properties of the oil. It was found that the addition of reduced graphene oxide results in worse anti-friction and anti-wear properties of the oil due to the presence of oxygen functional groups on the surface, compared to physical graphene without the presence of oxygen groups. The inferior lubricating properties of RGO are explained by the presence of oxygen-containing groups that increase the surface activity of the flakes, as well as many defects in the material and the contact surface [23]. Another study found that the lubricating properties of the base oil also depend on the number of layers and the size of the RGO particles. It was shown that the best tribological properties were obtained for graphene with a smaller flake size and containing a larger number of layers; the reduction of friction was 37% and wear was 47% lower compared to pure base oil [24].

Based on the literature review, it can be concluded that the addition of graphene to lubricants improves their tribological properties. However, graphene still remains the object of tribological research and the obtained results are not always unequivocal. This may result from the use of various forms of graphene (graphene oxide, reduced graphene oxide, graphene nanoplatelets), different sizes of flakes, different number of carbon layers, different content of oxygen functional groups on the surface and different media in which graphene and its derivatives are dispersed, such as also different test conditions. Therefore, the available information is still incomplete and requires further research. The complexity of the application of graphene in terms of improving the tribological properties of lubricants is also indicated by Larrson et al. [25]. Moreover, in the source literature there are significant discrepancies in the results of the research concerning the influence of graphene concentration in the base lubricant on its tribological properties. The literature lacks results of comparative studies on the effect of the type of graphene (e.g., graphene oxide, reduced graphene oxide, graphene nanoplatelets) on the properties of the lubricant. A very important issue is also the development of a method of introducing graphene into grease or oil in order to obtain a homogeneous dispersion of the nanofiller in the lubricant and, consequently, to improve the lubricity of the product. The literature discusses the problem of agglomeration of graphene flakes; in particular, this problem concerns lubricating oils [20].

Bearing in mind the above-mentioned limitations of the available literature data, this work proposes an innovative approach to the implementation of graphene materials as a nanoadditive with anti-wear properties to greases. For the purposes of the work, graphene materials were designed and manufactured, representing two types of flake graphene: graphene oxide and reduced graphene oxide. The paper presents the comparative results obtained for both materials. In order to obtain the best knowledge about the influence of graphene materials on the anti-wear properties of the grease, the tests were carried out for a wide range of concentrations of graphene materials in the grease, amounting to 0.25–5.00 wt.%. The experiment designed in this way allowed to obtain a detailed quantitative description of the issue under study. In addition, the article attempts to answer which of the two tested types of graphene materials will better meet the requirements for carbon nanoadditives for lubricants, taking into account their practical application.

## 2. Materials and Methods

**Figure 1:**
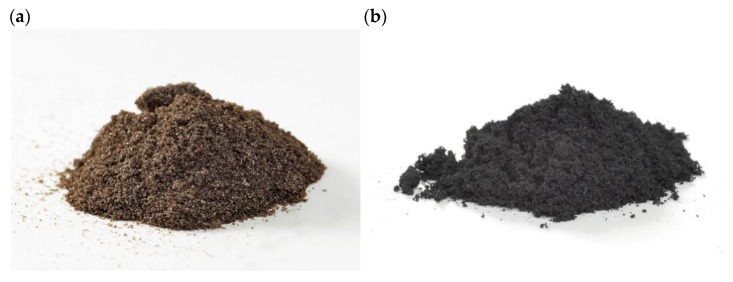
Graphene materials used in the tests: (**a**) graphene oxide, (**b**) reduced graphene oxide.

The research was conducted using graphene oxide powder and reduced graphene oxide powder by G-Flake^®^ (Łukasiewicz Research Network—Institute of Microelectronics and Photonics, Poland)—Figure 1.

Graphene oxide was produced using a modified Hummers method. This method is based on the oxidation of flake graphite (Asbury Carbons, Asbury, NJ, USA) in sulfuric acid VI (96%, pure p.a., Chempur, Poland) with the addition of potassium permanganate (pure p.a., Chempur, Piekary Śląskie, Poland) and potassium nitrate (pure p.a., Chempur, Poland). After the oxidation process, the graphite oxide in the form of an aqueous suspension is exfoliated and then purified by filtration. After the purification process, an aqueous suspension of graphene oxide is obtained. In order to produce reduced graphene oxide, graphene oxide undergoes a reduction reaction, which takes place at high a temperature in the presence of a reducing agent—hydrazine (pure p.a., Chempur, Piekary Śląskie, Poland). After the reduction process, the reduced graphene oxide is purified by filtration. To obtain GO or RGO in powder form, the aqueous material dispersion was dried. The obtained graphene materials (GO and RGO) were then characterized using the following methods: scanning electron microscopy (SEM) (Auriga^®^ CrossBeam^®^ Workstation, Carl Zeiss, Oberkochen, Germany, at a voltage of 1 kV, energy selective backscattered EsB detector), Raman spectroscopy (Renishaw Invia Raman Microscope, Wotton-under-Edge, UK, a laser with a wavelength of 532 nm was used for excitation), elemental analysis (628 Series and 836 Series, Leco Corporation, St. Joseph, MI, USA). Regarding the SEM experiment, it was implemented in order to visualize GO and RGO materials. The powders were placed on a silicon wafer and then glued with a carbon tape to an SEM table. No coating with conductive agent was conducted prior to visualization. As for the Raman examination, collected raw data were analyzed with Wire 3.54 software provided by the spectrometer producer. The analysis was carried out in order to discriminate the position of D and G peaks using a Gaussian/Lorentzian. Prior to determination of the peaks, position baseline subtraction was implemented. Detailed characterization of the purified GO water suspension, from which GO powder and RGO powder were then obtained, is presented in our earlier publication [26].

The tests of the lubricating properties of GO and RGO were carried out with the use of plastic grease. The plastic base lubricant was prepared using Finavestan A 360 B paraffin oil (pure, TOTAL, Gonfreville-l’Orcher, France) and lithium stearate (pure, ROTH, Karlsruhe, Germany) thickener. It was prepared in laboratory conditions by adding the thickener lithium stearate to the paraffinic base oil, mixing and then heating with constant stirring until the thickener completely dissolved (to approx. 220 °C). The dissolved mixture was allowed to cool completely at room temperature.

**Figure 2:**
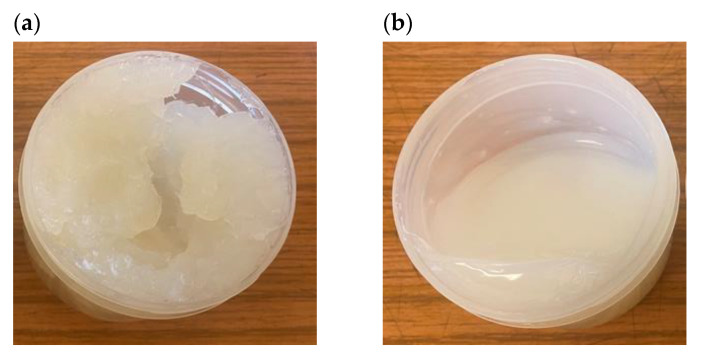
The appearance of the base grease: (**a**) before mixing; (**b**) after mixing in a planetary mixer.

The lubricant compositions were prepared in laboratory conditions by adding the appropriate amount of GO or RGO in the form of a powder to the lubricant base. The samples were then mixed using a planetary mixer (SpeedMixer DAC 150.1 FV-K, Hauschild Engineering, Hamm, Germany) to obtain a homogeneous dispersion (3500 rpm, 15 min). Mixtures of grease and flake graphene were subjected to tribological tests, in which the concentration of graphene (GO, RGO) was from 0.25 wt.% up to 5.00 wt.%. It was observed that as a result of the shear stresses arising when mixing pure base grease and base grease with the addition of graphene, the viscosity of the grease decreased due to the shear-thinning phenomenon (Figure 2).

The authors of the study [19] described analogous observations. The authors of this article have experimentally verified that the occurrence of this phenomenon does not affect the properties of the base lubricant and the obtained test results. On the other hand, the high shear stresses arising during the preparation of the mixtures prevent the agglomeration of graphene; therefore, it was possible to obtain a homogeneous dispersion of grease and graphene.

**Figure 3:**
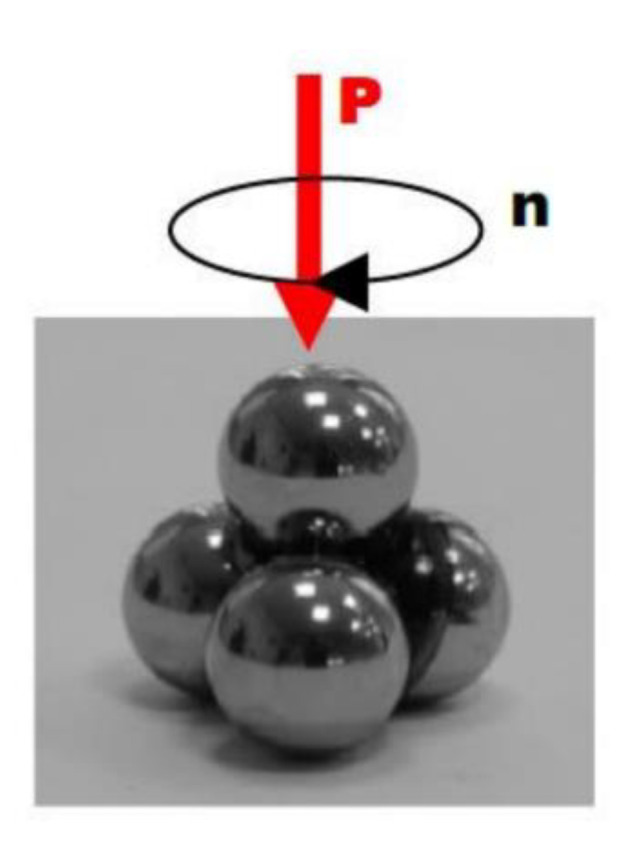
Diagram of a friction linkage in a 4-ball tester.

The tests of the lubricating properties of the obtained plastic greases with the addition of flake graphene were determined by measuring the average diameter of the wear scar. The tests were performed with the use of a 4-ball tester (Stanhope-SETA Limited, Chertsey, UK). In this tester, the elements of the tribological couple are in point contact and a sliding movement is performed. The tribological couple in the tester consists of four balls with a diameter of 12.7 mm, made of bearing steel with a hardness of 62.7 HRC. Three balls (stationary) are placed in the lower holder, into which the tested lubricant is also introduced (in the amount of 8 ± 2 cm^3^). These balls are pressed down with a ring and fixed with a tightened clamping nut. The fourth ball (movable) is mounted in the upper holder. During the test, it rotates at a speed of 1450 ± 50 rpm, which corresponds to the speed of the rubbing surfaces of 0.55 m·s^−1^. The balls in the lower holder are pressed with the force P against the ball mounted in the upper holder by means of a lever. The friction linkage diagram is shown in Figure 3.

**Figure 4:**
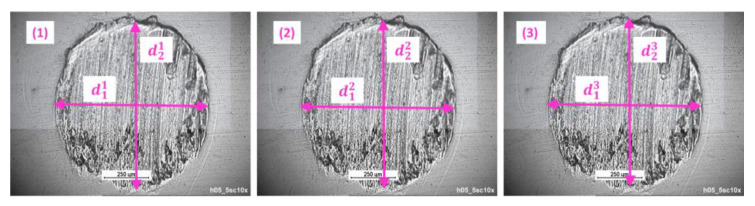
The principle of calculating the average diameter of the wear scar in a given test run: (**1**) the wear scar on the first ball, (**2**) the wear scar on the second ball, (**3**) the wear scar on the third ball.

Before starting the tests, the balls were washed in gasoline and, after drying, stored in acetone. At the end of each run, the stationary balls were washed in extractive gasoline. At least three test cycles were carried out for each lubricant. After the tribological tests, the diameter of the friction scars formed on the test elements was measured. The diameter was measured on three balls from a given run, perpendicular ($d_{1}$) and parallel ($d_{2}$) to the friction scars, and then the arithmetic mean ($\bar{d}$) was calculated, which was taken as the mean track diameter in a given test run. The principle of calculating the average diameter of the wear scar is shown in Figure 4 and Equation (1).

(1) $$\bar{d}=\frac{d_{1}^{1}+d_{2}^{1}+d_{1}^{2}+d_{2}^{2}+d_{1}^{3}+d_{2}^{3}}{6}$$

The size of the trace diameter was the basic measure of the tribological properties of the tested substances. The arithmetic mean of the results of at least three determinations which did not differ from their arithmetic mean by more than 10% was taken as the result of the determination. During the research work with the use of a 4-ball tester, two types of tests were carried out: at a load of 60 kgf for 60 min; at a load of 100 kgf for 1 min. The adopted conditions result from the internal procedure for testing anti-wear additives.

## 3. Results and Discussion

### 3.1. Characterization of Flake Graphene

**Figure 5:**
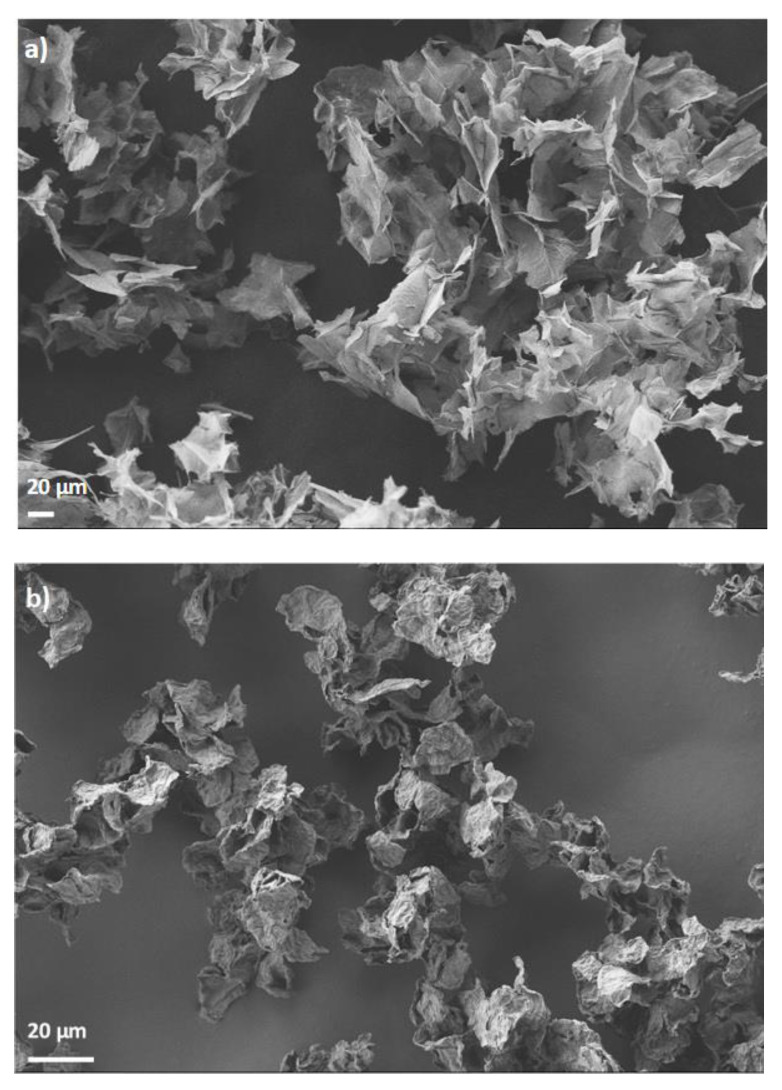
SEM images of (**a**) graphene oxide, (**b**) reduced graphene oxide.

Figure 5 shows SEM images of graphene oxide and reduced graphene oxide. Figure 5a shows GO in the form of a powder, the size of the structures is 5–30 µm, the flake size of GO in the aqueous suspension from which the powder was obtained is 2–10 µm [26] (drying causes agglomerates of graphene flakes). The SEM image of the RGO powder is shown in Figure 5b; the size of the structures is 5–20 µm.

**Figure 6:**
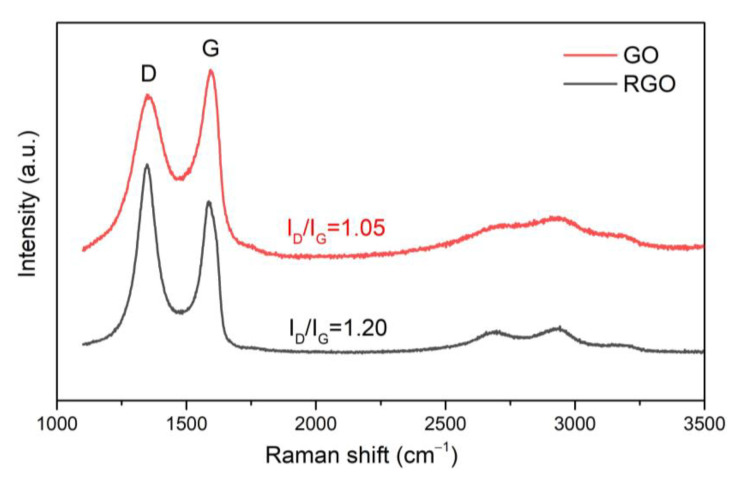
Raman spectra of graphene oxide and reduced graphene oxide.

Figure 6 shows the Raman spectra for both tested graphene derivatives GO and RGO. Raman spectroscopy was used to estimate the degree of reduction of GO and to define structural differences between GO and RGO. Raman spectroscopy has been proven to be useful for detailed research description of graphene materials [27,28]. The Raman spectrum of carbon materials contains bands marked as D and G. The D band (located near 1350 cm^−1^) results from the presence of vacancies or dislocations in the graphene layer and at the edge of this layer. This band is also related to the presence of defects in the material. The G band (located near 1590 cm^−1^) corresponds to the *sp*^2^ hybridization of the carbon network and is attributed to the first-order scattering from the doubly degenerate E2g phonon modes of graphite in the Brillouin zone center as well as bond stretching of *sp*^2^ carbon pairs in both rings and chains [29]. The ID/IG ratio is related to the amount of defects present in the material [30]. The analysis of Raman spectra for both materials (Figure 6)—GO and RGO—indicates the presence of an intense peak D at a length of ~1357 cm^−1^ for GO and ~1347 cm^−1^ for RGO, respectively, and a G peak at ~1586 cm^−1^. The ID/IG ratio for GO is 1.05 and for RGO 1.20. The value of the ID/IG parameter increases with the increase in the degree of disorder in the structure of the graphene material.

The results of elemental analysis of graphene materials are presented in Table 1. The oxygen content in GO is 45.0–52.0%, while in the case of RGO it is 15.0–20.0%. The carbon content is 40.0–42.0% for GO and 70.0–80.0% for RGO. The content of the other elements—sulfur, nitrogen and hydrogen—is less than 3.0%.

### 3.2. Anti-Wear Properties of the Grease

**Figure 7:**
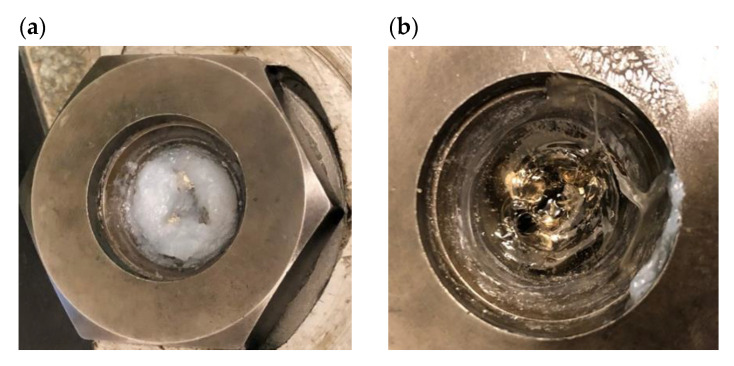
Base grease in the lower handle of the 4-ball tester: (**a**) before and (**b**) after the tribological test-node load 100 kgf for 1 min.

The grease is a colloidal system, where the dispersing phase is oil and the dispersed phase is thickeners. The role of the thickener is to ensure the proper spatial structure of the plastic grease and the specific, required rheological and tribological properties. The spatial structure formed in the lubricant by the thickener fibers, as a result of the action of shear forces and the temperature increase during the operation of the tribological node, disintegrates [31,32]. An example of the appearance of the base grease in the lower holder of the 4-ball tester before and after the test (load 100 kgf, time 1 min) is shown in Figure 7. The lubricant in the mating contact changes its structure from semi-liquid (Figure 7a) to liquid (Figure 7b) as a result of shear forces and an increase in temperature in the contact zone of friction elements. The grease is non-Newtonian liquid, classified as thixotropic liquid. Under the influence of shear forces, its internal structure is destroyed and viscosity is reduced [33]. Oil is separated as a result of breaking the bonds of the cross-linker, which enables better lubrication.

**Figure 8:**
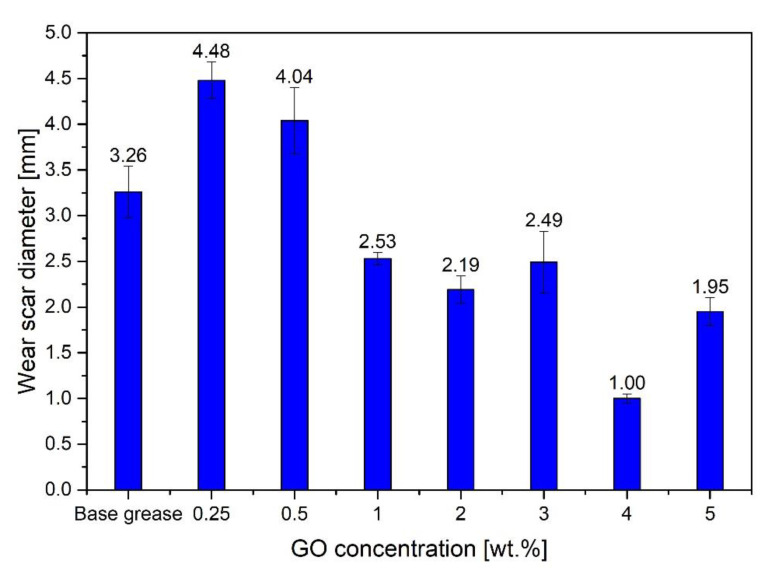
Influence of the concentration of GO additive on the tribological properties of a lubricant.

Prepared lubricants with different contents of graphene material (GO, RGO) were subjected to anti-wear tests. Figure 8 shows the results of studies on the effect of GO concentration in the range of 0.25–5.00 wt.%. on lubricating properties, for a load of 60 kgf for 60 min. The obtained data show that the introduction of GO to the base lubricant in a small concentration of 0.25 wt.%. and 0.50 wt.%, increases the wear of the test pieces by approx. 37% (4.48 ± 0.20 mm) and 24% (4.04 ± 0.36 mm), respectively, in relation to the base lubricant without additive (3.26 ± 0.28 mm). However, the introduction of a GO additive in an amount of 1.00 wt.% results in a significant improvement in lubricating properties and reduction of wear. Then, as the concentration of GO in the lubricant increases from 1.00 wt.% to 4.00 wt.%, the degree of reduction of the average wear scar diameter increases from 22% (2.53 ± 0.07 mm) to 69% (1.00 ± 0.05 mm). A reduction value of the average wear scar diameter for a concentration of 3.00 wt.% was similar to 1.00 wt.%, amounting to approx. 24%. Further increase of the GO concentration to 5.00 wt.% results in a re-increase in the average diameter of the wear scar, but still the degree of wear is 40% less compared to the base grease

**Figure 9:**
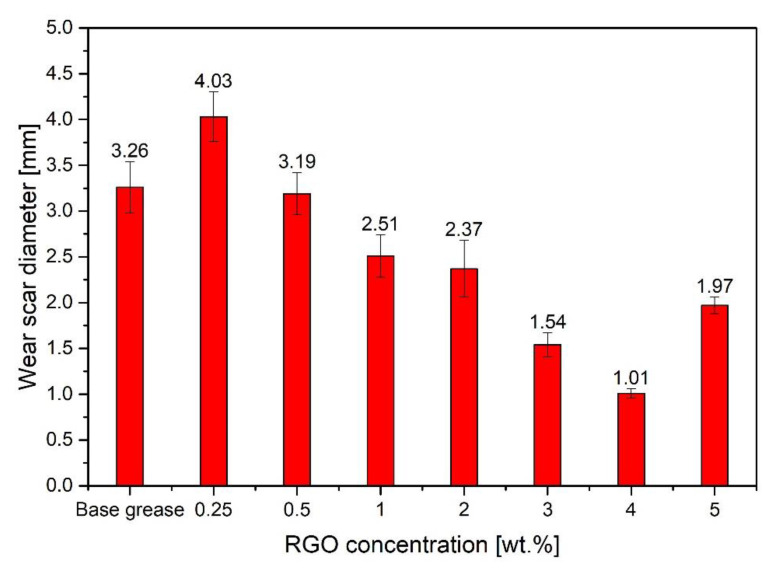
Influence of RGO additive concentration on the tribological properties of a lubricant.

Figure 9 shows the results of the research on the effect of the concentration of the second form of grapheme—RGO (0.25–5.00%)—on the lubricating properties, for a load of 60 kgf for 60 min.

In the case of lubricant samples containing the additive in the form of RGO, the trend observed was similar to the compositions with the addition of GO. In this case, an increase in the wear of the test elements by 24% (4.03 ± 0.27 mm) was also observed compared to the base lubricant (3.26 ± 0.28 mm) for the lowest additive content of 0.25 wt.%. Increasing the concentration of graphene to 0.50 wt.% results in a wear scar with an average diameter close to the scar obtained when lubricating the node with base grease without additive. Only higher concentrations of the nanoadditive result in a significant reduction in consumption. The lowest wear was observed at 4.00 wt.%. RGO concentration (scar reduction by approx. 70%). Increase in RGO concentration in the lubricant from 1.00 to 4.00 wt.% reduces the average diameter of the wear scar from 2.51 ± 0.23 mm (23%) to 1.01 ± 0.05 mm (69%), respectively, compared to the grease without additive. Further increase of the additive content to 5.00 wt.% may increase the wear of the balls (1.97 ± 0.09 mm). It can be concluded that the RGO concentration of 4.00 wt.% in the grease is the limit concentration, exceeding which the antiwear properties of the grease may deteriorate. Even though 5.00 wt.% RGO concentration increases the consumption in relation to the concentration of 4.00 wt.%, in relation to the grease without the addition of graphene, the average diameter of the wear scar was reduced by 40%. Deterioration of lubricating properties for the highest concentration of the additive 5.00 wt.% has also been observed as a result of the use of GO. In the case of high concentrations of graphene, agglomerates form in the lubricant, which negatively affect the friction surface protection. The resulting tendency to change the wear properties of a lubricant with an increase in graphene concentration is consistent with the results of the research carried out by Fu et al. [20]. According to the published results, with an increase in the concentration of graphene nanoplatelets from 1.00 wt.% to 2.00 wt.%, the diameter of the wear scar decreases, reaching the lowest value for the 2.00 wt.% concentration. Further increase in graphene concentration to 4.00 wt.% results in deterioration of the anti-wear properties of the lubricant in relation to the concentration of 2.00 wt.% [20]. The difference in the concentration range for which the scar reduction is observed may result from the properties of the graphene material, the type of lubricant base, and the test conditions. By the way, it should be added that adding RGO to the lubricant, apart from improving the tribological properties of the lubricant, may also increase the efficiency of heat transfer from the friction junction, which will also have a positive effect on the junction’s operation. This is due to the fact that this material has good thermal conductivity [34,35]. Fu et al. reported, that the addition of graphene into the base grease effectively enhances the tribological properties and thermal conductivity. The thermal conductivity of the grease with 4.00 wt.% graphene reached 0.28 W/mK, which is an increase of 55% compared to the base grease [20]. As a result of the research, it was observed that the viscosity of the lubricant increases with the increase in the concentration of GO or RGO in the lubricant, as in the study [20]. Lubricants with both GO and RGO additions also thicken during friction and locally near the ball contact, compared to the base grease. The graphene agglomerates are then visible in the lubricant. Grease is a multi-phase material, where the base oil is trapped within the thickener network by a combination of van der Waals and capillary forces. Their rheology under the influence of frictional forces is still not well understood and remains the subject of research. The properties of the lubricant base depend on the type of oil and thickener and the introduction of an additive further complicates the interactions between the lubricant and the protected surface. The mechanism of interaction between lubricant and graphene molecules is still unknown. In addition, high surface activity, in particular GO, but also RGO resulting from the presence of oxygen functional groups may cause a violation of the internal structure of the lubricant and may cause reduced wear protection at low additive contents, which results in a larger wear scar diameter for concentrations in the range of 0.25–0.50 wt.% compared to pure base grease. The concentration of graphene in the lubricant is probably too low to compensate for the breakdown of the lubricant’s internal network. This effect is more evident in the case of the lubricant with the addition of GO; it is indicated by the worse results obtained for the concentration of GO 0.25–0.50 wt.%, compared to RGO in the same concentrations. Graphene oxide has much more oxygen functional groups compared to RGO, which increase its activity. The role of oxygen functional groups in RGO for tribological properties of polyethylene glycol (PEG) was investigated by Gupta et al. [36]. Moreover, it may be that individual graphene flakes spread over the surface make it difficult for the lubricant to reach the surface interface, resulting in increased wear. Only higher concentrations of graphene flakes are able to cover enough of the friction surface to protect the node elements. This effect is noticed up to a concentration of about 4.00 wt.%. Probably, at this concentration the protective layer formed by graphene flakes is thick enough to be the most resistant to the forces that occur during tests at the friction node. At the same time, this amount of graphene does not adversely affect the density of the lubricant so much that it makes it difficult to protect the surface. Concentrations above 4.00 wt.% result in greater wear, which may be due, among other things, to the noticeable increase in lubricant density after the introduction of such an amount of additive. However, the observed phenomenon requires further, more detailed research.

During the tests of lubricants, the color of the lubricant with the addition of GO changed from brown to clearly darker, which may suggest that the graphene oxide is reduced during friction [37,38]. The reduction process may take place under the influence of the temperature increase of the lubricant in the friction node or tribochemical reactions. Moreover, as a result of the reduction of GO oxygen groups, gaseous products are formed [39], which may additionally adversely affect the lubricating properties of the product and the surface of the friction junction. Therefore, it is advisable to use RGO as an additive to the lubricant instead of GO. The use of RGO ensures the stability of the lubricant composition during operation in the friction node, while maintaining the same lubricating properties as in the case of the GO additive.

**Figure 10:**
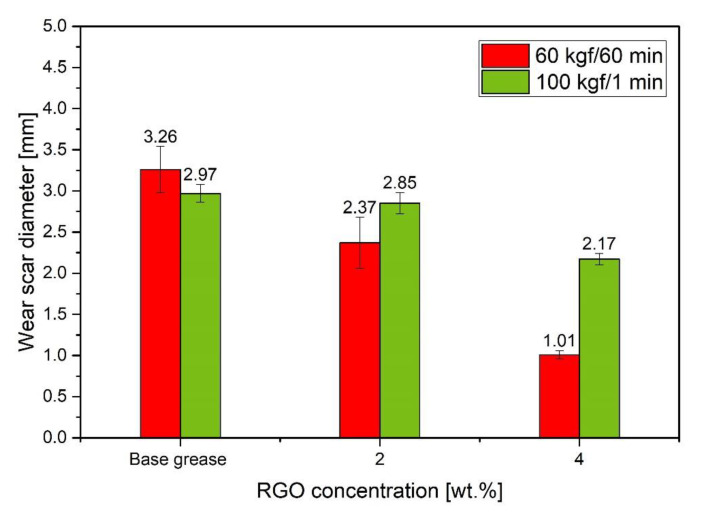
Average diameter of the wear scar of grease with RGO additive depending on the load (60 kgf for 60 min and 100 kgf for 1 min).

Tests were also carried out on lubricants with the addition of RGO for a higher load of 100 kgf for 1 min and compared with the results obtained in the tests with a load of 60 kgf and a duration of 60 min, and the results of the measurements are presented in Figure 10. The obtained data indicate that in the case of tests with higher loads (100 kgf, 1 min), the introduction of the RGO additive causes a gradual reduction of the wear scar along with the increase in concentration from 2.00 wt.% to 4.00 wt.%; the reduction was 4% and 27%, respectively. The performance of the test under milder conditions translates into a greater reduction of the wear scar, which is particularly noticeable for the RGO concentration of 4.00 wt.%. Then the reduction of the wear pattern in relation to the base grease at a load of 100 kgf is 27% (2.17 ± 0.07 mm) and at 60 kgf as much as 69% (1.01 mm ± 0.05), compared to pure base grease. This may indicate the anti-wear rather than anti-seizure character of the protective layers created by the nanoadditive.

## 4. Conclusions

Grease samples were produced with the addition of GO and RGO with a concentration in the range of 0.25–5.00 wt.%. It was found that the addition of flake graphene improves the anti-wear properties of the lubricant, while the concentration and type of graphene have an impact on the tested lubricant properties and its stability. For lower contents of graphene additive (0.25 wt.% and 0.50 wt.% for GO and 0.25 wt.% for RGO), an increase in wear is observed as compared to the base grease. For low RGO concentrations (0.50 wt.%), the grease properties are similar to those of the base grease without additive. The introduction of flake graphene to the base of the plastic grease may disturb the internal network of the grease due to the high activity of the surface of the graphene flakes. This phenomenon may be the cause of increased node wear at low additive concentrations (0.25 wt.% and 0.50 wt.%), as these contents are probably too low to compensate for the breakdown of the lubricant’s inner network. This effect is particularly evident in GO containing numerous oxygen functional groups on the surface of the flakes.

The use of a higher concentration of graphene 1.00–5.00 wt.% results in a reduction in the average diameter of the wear scar, which is greatest for an additive content of 4.00 wt.% (reduction of the wear scar diameter was 69% for GO, as well as for RGO). A further increase in the concentration of the additive to 5.00 wt.% results in a renewed increase in wear, which may result from the agglomeration of the graphene material and a significant increase in viscosity, so that the lubricant is not able to produce a lubricating film that will protect the friction surface well.

Out of the two tested forms of flake graphene, RGO is a recommended additive for lubricants due to its stability under operating conditions, while GO was reduced during friction. The RGO powder tested in operation can therefore be successfully used as a material improving the tribological properties of lubricants.

The use of flake graphene in lubricants is not a thoroughly researched and explained issue. In order to further develop the application of graphene flakes as a lubricant additive, it is necessary to continue research, including in the direction of chemical modification of the flake surface, and to explain the mechanisms of graphene operation during friction, and also to maintain the properties of lubricants unchanged in subsequent work cycles.

## Figures and Tables

**Table 1:** Elemental analysis of graphene oxide and reduced graphene oxide.

	Composition (%)				
	C	O	S	N	H
GO	40.0–42.0	45.0–52.0	1.0–3.0	<0.3	2.0–3.0
RGO	70.0–80.0	15.0–20.0	<2.0	<0.3	<0.2

## Data Availability

Data are contained within the article.
